# A Rare Case of Rash, Blurry Vision, and Papilledema Caused by Isolated Ocular Syphilis

**DOI:** 10.7759/cureus.72371

**Published:** 2024-10-25

**Authors:** Joshua Faber, Emily Cen, Vinay Saggar

**Affiliations:** 1 Emergency Medicine, Columbia University College of Physicians and Surgeons, New York, USA

**Keywords:** blurry vision, ocular syphilis, papilledema, point-of-care ultrasound (pocus), syphilitic papillitis

## Abstract

Papilledema is a high-risk cause of vision changes in the Emergency Department (ED) and a critical physical examination finding because of its close association with etiologies that may progress to vision loss or death. Syphilis is a rare infectious cause of papilledema, with scarce case reports published showing its ability to develop such sequela. We present a case of a 35-year-old male with a past medical history of newly diagnosed HIV who originally presented to the ED with a rash and rapidly worsening visional changes. While in the ED, the patient developed a brief syncopal episode and acute vision loss, after which concern for increased intracranial pressure and possible opportunistic infection guided the clinicians to perform a bedside point of care ultrasound (POCUS) that confirmed bilateral papilledema. The patient ultimately had an expedited work-up that rendered a diagnosis of syphilitic papillitis and uveitis without associated neurosyphilis. With the growing incidence of syphilis in the US, we aim to shed light on the rare diagnosis of isolated ocular syphilis in hopes of increasing awareness of this potentially vision-threatening pathology. This case also serves as an important reminder of the utility of POCUS in working up undifferentiated visual complaints and concerns of papilledema.

## Introduction

Papilledema is the optic disc swelling due to increased intracranial pressure [1]. It is a critical physical examination finding to screen for in the Emergency Department (ED), particularly for patients who present with neurologic or visual complaints because of its close association with etiologies that can progress to complete vision loss or death. The differential for why a patient may have acute papilledema is broad, including vascular dysfunction, infection, neoplasm, and more [1]. While a thorough physical exam can help narrow the diagnosis, Emergency Medicine Physicians (EPs) often may not have the proficiency, time, or resources needed to perform a comprehensive fundoscopic exam to evaluate this key finding [2]. Fortunately, studies have continued to support the use of prompt point-of-care ultrasound (POCUS) as a suitable surrogate for identifying papilledema and other fundoscopic findings [3]. The sensitivity and specificity of diagnosing papilledema on POCUS have been shown to range from 70-90% and 69-100%, respectively [4].

Ocular syphilis is a rare infectious cause of papilledema. Syphilitic uveitis is the most common cause of ocular syphilis, with one recent study estimating the incidence at 0.15 per 100,000 people in the United States (US) [5]. Risk factors include male sex, African American race or Hispanic ethnicity, and a medical history of acquired immunodeficiency syndrome (AIDS), and symptoms are often non-descript, including blurry vision, vision loss, and eye pain [6].

We present a case of a 35-year-old male with a past medical history of newly diagnosed HIV who presented to the ED with a rash and rapidly worsening visional changes, ultimately having papilledema on POCUS and an expedited work-up that rendered a diagnosis of isolated ocular syphilis without associated neurosyphilis.

## Case presentation

A 35-year-old male with newly diagnosed HIV presented to the ED with rash and visual changes. The patient reports he had visited an outside hospital three days prior due to a pruritic rash, where he was diagnosed with urticaria and discharged on antihistamines. Two days later, the patient states he began developing chills and generalized joint pain, where he was found to have HIV but left before completion of his work-up. Within less than 24 hours, he presented to our ED. 

On initial presentation, the patient endorsed a persistent rash and rapidly developing bilateral, left greater than right blurry vision. Physical exam was remarkable for a blanching maculopapular rash on bilateral upper and lower extremities, bilateral inferior hemianopsia, and an afferent pupillary defect of the left eye that was not present on the right. While awaiting further work-up, he witnessed a syncopal episode. He quickly returned to baseline and had repeat vitals within normal limits.

**Figure 1:**
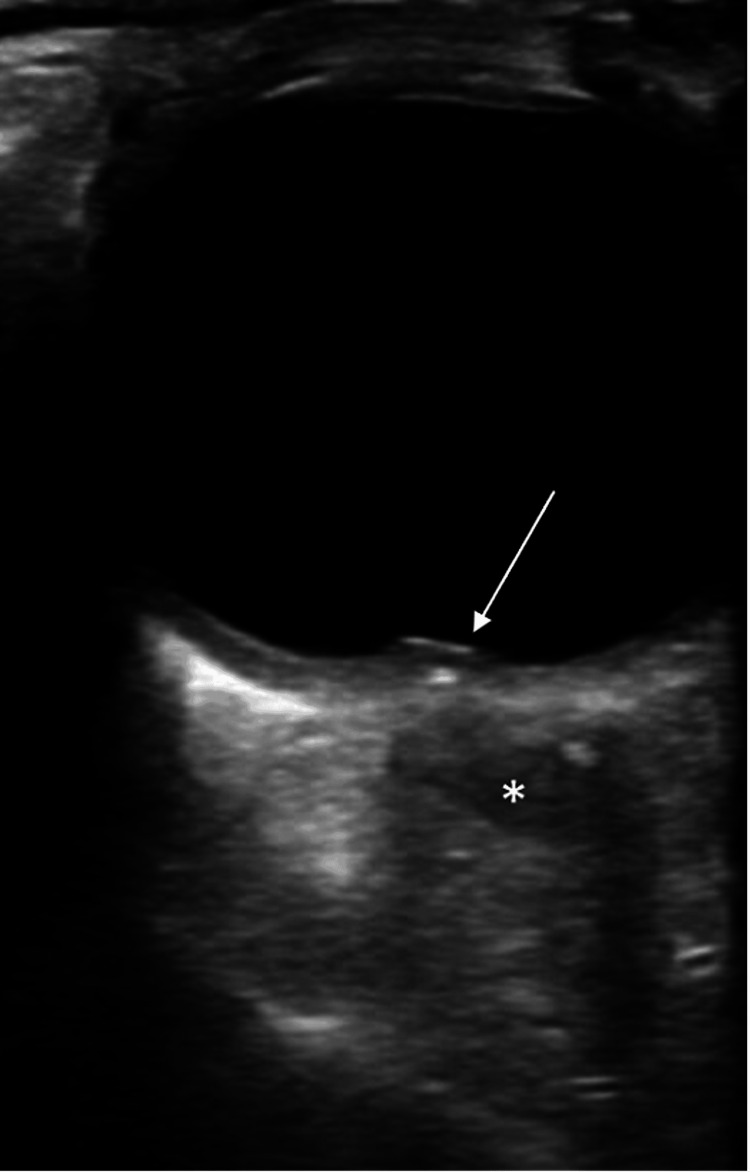
Point of care ocular ultrasound Ocular ultrasound demonstrating optic disc elevation (arrow) with a dilated, hypoechoic optic nerve sheath (*) just posteriorly, suggestive of increased intracranial pressure and papilledema

**Figure 2:**
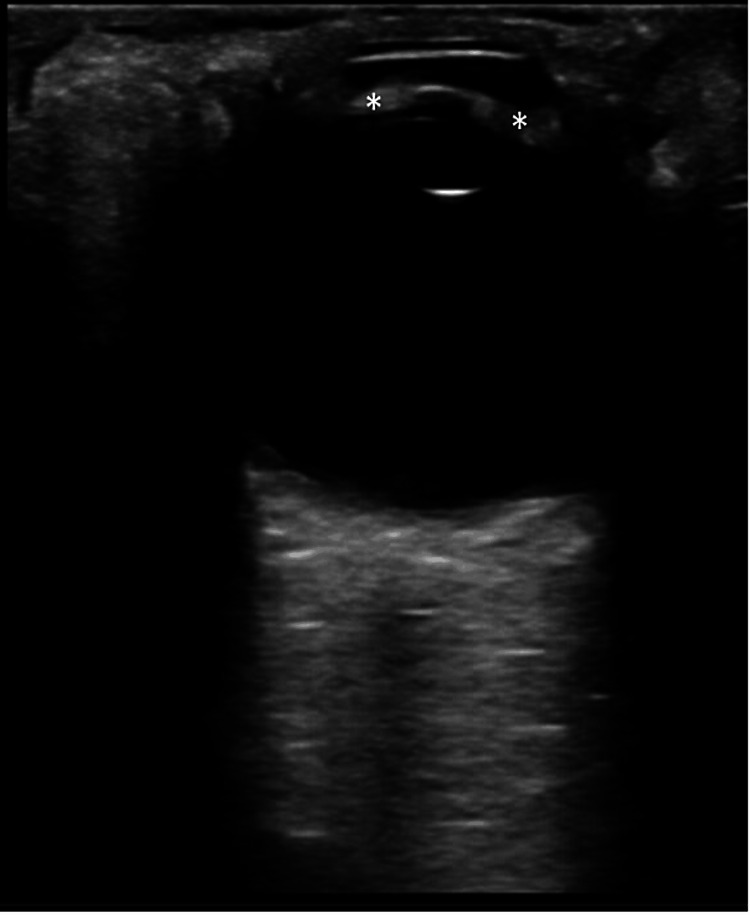
Point of care ocular ultrasound Ocular ultrasound showing the uvea(*), which appears thickened

Given the patient's comorbidity and these signs and symptoms, there was a heightened level of concern for possible opportunistic infection and increased intracranial pressure. While initial blood work was being drawn and expedited, non-contrast computed tomography (CT) of his head was ordered. A rapid bedside Point of Care Ultrasound (POCUS) was done to screen for increased intracranial pressure and papilledema. The point-of-care ocular ultrasound revealed a dilated optic nerve sheath suggestive of increased intracranial pressure (Figure 1), elevation of the optic disc suggestive of papilledema (Figure 1), and a grossly thickened uvea (Figure 2). Ophthalmology was then immediately consulted.

Initial blood work, including a complete metabolic panel, complete blood count, and point-of-care venous blood gas, was unremarkable. The patient had a negative, expedited, non-contrast computed tomography (CT) of his head.

**Figure 3:**
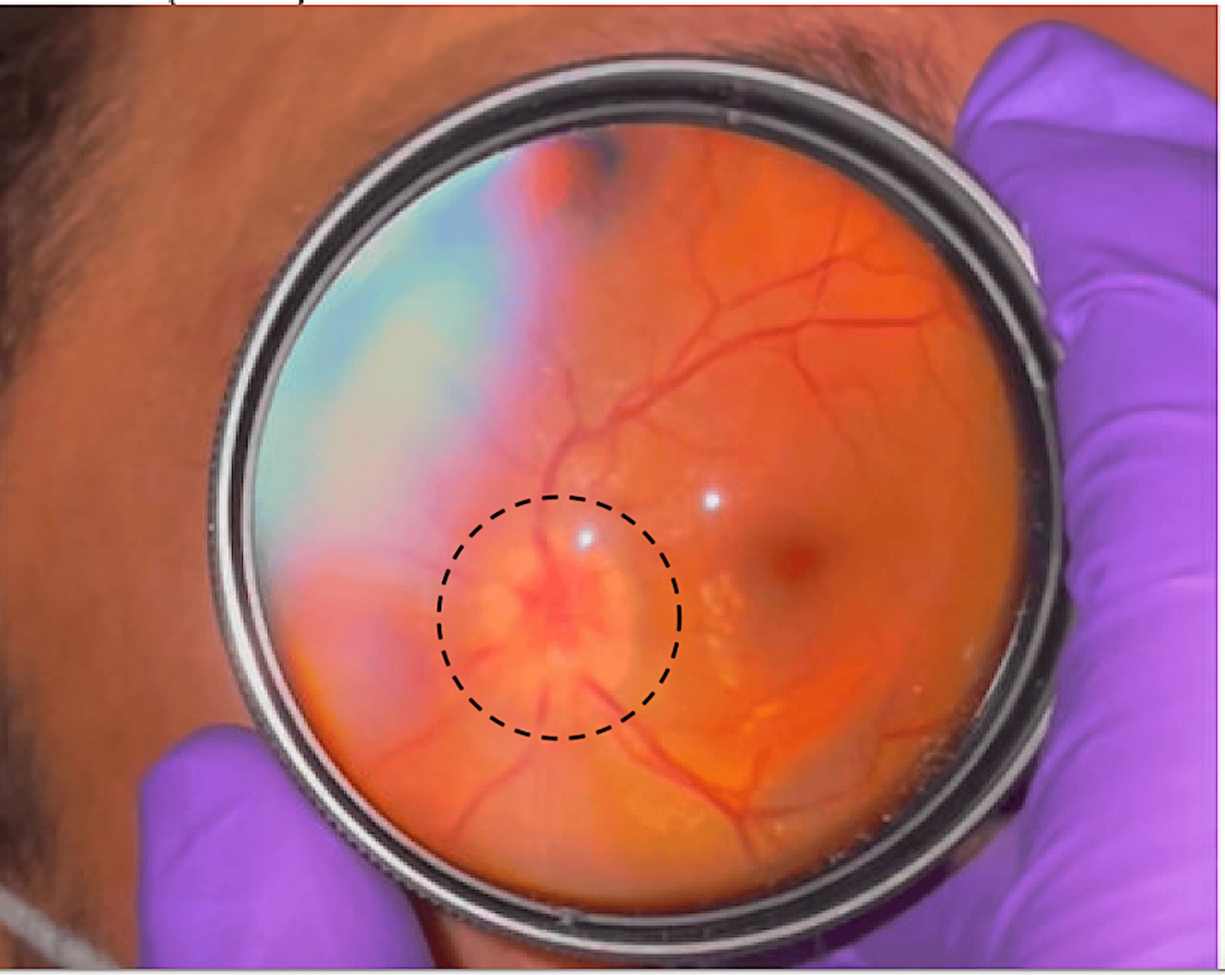
Fundoscopic findings A dilated fundoscopic exam performed by ophthalmology revealed bilateral optic disc edema (circle) suggestive of papilledema, without disc hemorrhage

A dilated fundoscopic exam performed by the ophthalmologist showed bilateral optic disc edema (Figure 3), suggestive of papilledema, without disc hemorrhage. A dedicated ocular exam also found visual field deficits in the bilateral inferonasal aspect of both eyes, the inferotemporal area of his left eye, and a left relative afferent pupillary defect with 20/800 vision. The patient was seen by neurology, who requested magnetic resonance imaging (MRI) of his brain due to bilateral disc swelling seen on the exam and recommended a broad infectious work-up, including assays for toxoplasma, cytomegalovirus, herpes simplex virus, syphilis, tuberculosis, and Lyme. The patient was admitted to the hospital under the neurology service.

**Figure 4:**
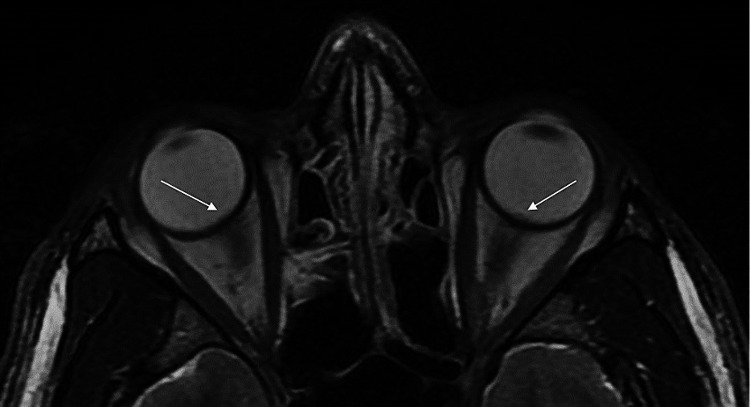
MRI T2-weighted sequencing of the bilateral orbits MRI image showing protrusion of bilateral optic nerves into the globes consistent with papilledema (arrows)

During day one of admission, his broad infectious work-up was only positive for reactive serum RPR, and his MRI Brain with and without contrast showed isolated protrusions of the bilateral optic nerve heads into the posterior globes consistent with papilledema (Figure 4). The patient underwent lumbar puncture with normal cerebrospinal fluid studies and was ultimately diagnosed with isolated intraocular syphilis. Throughout his hospitalization, the patient was started on intravenous penicillin, bictegravir/emtricitabine/tenofovir alafenamide, and trimethoprim/sulfamethoxazole with an eventual improvement of vision in the left eye to 20/50 as well as improvement of a notable afferent pupillary defect in the left eye. The patient was ultimately discharged with a follow-up to the ophthalmology and uveitis eye clinic four days after admission.

## Discussion

The first implication of this case report is the heterogeneity in ocular syphilis pathophysiology. Syphilis has become more common over the past five years. According to the CDC, rates nationally increased by 78.9% from 2018 through 2022 [7]. However, ocular syphilis remains rare as a diagnosis. Reviewing the literature, very few case studies published since 2020 highlight bilateral syphilitic papillitis [8-11]. Of those cases, two involved diagnosed neurosyphilis, while two demonstrated non-reactive CSF VDRL. In this case study, all of the patient's imaging studies confirmed papilledema, but lumbar puncture (LP) opening pressure was normal, and therefore, the patient was eventually diagnosed with bilateral papillitis without neurosyphilis. In general, the epidemiology of syphilitic papillitis versus true papilledema is poorly understood due to their rarity; however, it may be worth revisiting in light of increasing ocular syphilis rates [12].

Second, this case highlights the utility of POCUS in working up potential causes of blurred vision. Although a common ED complaint, visual disturbances can often be a challenging presentation for emergency physicians, as many providers may not have the time or expertise to accurately obtain and interpret the findings of a comprehensive fundoscopic examination [2]. POCUS offers a fast, convenient modality for evaluating blurred vision, and literature supports its ability to aid in the diagnosis of retinal and vitreous hemorrhage/detachments, ocular infections, foreign bodies, retrobulbar hematomas, papilledema or other vascular pathologies [3]. Specific to this case, POCUS was used to screen for increased intracranial pressure and papilledema. The most widely used and validated methods for evaluating these pathologies include measuring the hypoechoic optic nerve sheath diameter 3 mm behind the eye, where a length of 5 mm or greater is considered abnormal, and visually observing for optic disc elevation into the vitreous cavity [3]. POCUS, in this case, was able to reach the same conclusion as a fundoscopic exam performed by an ophthalmologist 2 hours sooner and yielded the benefits of an expedited work-up, including the ordering of advanced imaging (non-contrast CT head) and consultation of additional services (ophthalmology). Although the ophthalmologic literature around the diagnosis of ocular syphilis does not advance POCUS as an important first step in emergency department work-up, emergency medicine literature does, and this case report continues to support that stance [13-15].

Finally, this case report also demonstrates the heterogeneity in treatment paradigms for ocular syphilis. While syphilitic papillitis is traditionally viewed as responsive to steroids, they were not administered in this patient as his symptoms drastically improved with antibiotic therapy that was initiated before a successful LP and formal diagnosis of papillitis. Indeed, despite steroids not being given, his vision improved drastically from 20/800 to 20/50 from the left eye before discharge, along with a nearly resolved left afferent pupillary defect. As ocular syphilis rates increase, practitioners need to be aware that there is no "one size fits all" approach to disease management.

## Conclusions

This case was one of a patient who originally presented to the ED with rash and vision changes and was found to have bilateral syphilitic papillitis and uveitis. His medical history of newly diagnosed HIV, gender, and race were all risk factors for this pathology, and this case is particularly unique in being diagnosed with isolated ocular syphilis without associated neurosyphilis. The case also serves as an important reminder of the utility of POCUS in working up undifferentiated visual complaints. With the rising rates of patients being diagnosed with syphilis and the commonality with which emergency medicine providers see symptoms related to papilledema, awareness should be raised on the possibility of this vision-threatening pathology.

## References

[1] Chen JJ, Bhatti MT (2019). Papilledema. Int Ophthalmol Clin.

[2] Mackay DD, Garza PS, Bruce BB, Newman NJ, Biousse V (2015). The demise of direct ophthalmoscopy: A modern clinical challenge. Neurol Clin Pract.

[3] Kilker BA, Holst JM, Hoffmann B (2014). Bedside ocular ultrasound in the emergency department. Eur J Emerg Med.

[4] Ghanem G, Haase D, Brzezinski A, Ogawa R, Asachi P, Chiem A (2023). Ultrasound detected increase in optic disk height to identify elevated intracranial pressure: A systematic review. Ultrasound J.

[5] Mir TA, Kim SJ, Fang W, Harvey J, Hinkle DM (2024). Rising incidence of syphilitic uveitis-related hospitalizations in the US. JAMA Ophthalmol.

[6] Oliver SE, Aubin M, Atwell L (2016). Ocular syphilis - eight jurisdictions, United States, 2014-2015. MMWR Morb Mortal Wkly Rep.

[7] (2024). Sexually transmitted infections surveillance, 2022. https://www.cdc.gov/std/statistics/2022/default.htm.

[8] Buscho SE, Ishihara R, Gupta PK, Mopuru R (2022). Secondary syphilis presenting as bilateral simultaneous papillitis in an immunocompetent individual. Cureus.

[9] Fensterwald M, Oh A, Shieh P, Spiegel S (2022). An unusual case of isolated bilateral papillitis. Neurology.

[10] Zhou XY, Sobol WM (2022). Bilateral papillitis as the initial presentation of neurosyphilis in a patient previously treated for primary and secondary syphilis. Am J Ophthalmol Case Rep.

[11] Gonzalez-Martinez A, Quintas S, Vivancos DC, Cebrián J, Vivancos J (2020). Diagnosis of syphilitic bilateral papillitis mimicking papilloedema. Emerg Infect Dis.

[12] Tucker JD, Li JZ, Robbins GK (2011). Ocular syphilis among HIV-infected patients: A systematic analysis of the literature. Sex Transm Infect.

[13] Gutierrez-Luke S, Wolf T, Green K, Graber P (2024). A case report of unilateral syphilitic uveitis: A diagnostic challenge and the role of point-of-care ultrasound. Clin Pract Cases Emerg Med.

[14] Schulz DC, Orr SM, Johnstone R, Devlin MK, Sheidow TG, Bursztyn LL (2021). The many faces of ocular syphilis: Case-based update on recognition, diagnosis, and treatment. Can J Ophthalmol.

[15] Silva MS, Arantes TE, Moreto R, Smith JR, Furtado JM (2023). Vision-related quality of life in patients treated for ocular syphilis. Sci Rep.

